# Risk factors for motor decline following parasagittal and falx meningioma resection in the middle third

**DOI:** 10.3389/fonc.2025.1458934

**Published:** 2025-02-04

**Authors:** Chengxuan Guo, Yafei Xue, Yang Li, Qilong Tian, Yan Qu, Qing Cai

**Affiliations:** Department of Neurosurgery, Tangdu Hospital, Air Force Medical University, Xi’an, China

**Keywords:** middle third, falx meningioma, motor decline, risk factor, parasagittal meningiomas

## Abstract

**Objective:**

The resection of parasagittal and falx meningiomas in the middle third superior sagittal sinus (SSS) is associated with a high risk of postoperative motor deficits. This study discusses the risk factors affecting postoperative motor decline and recovery of motor function after follow-up.

**Methods:**

The results of a single-institution retrospective cohort study of parasagittal and falx meningioma resection in the middle third between 2016 and 2023 were reviewed, and parameters were screened as potential predictors. Variables of interest included postoperative motor decline and outcome after follow-up. Univariate and multivariate analyses were performed to identify risk factors.

**Results:**

Among 87 patients who underwent resection of parasagittal (63.2%) or falx (36.8%) middle third meningiomas, 14.9% (13/87) experienced postoperative motor decline. Among the 36 patients (41.4%) with preoperative motor deficits, 66.3% improved, 17.0% unchanged, and 16.7% (6/36) worsened following surgery. Among the 51 patients without preoperative motor deficits, 13.7% (7/51) developed new weakness. The predictors of postoperative motor decline were major venous involvement (p = 0.022), falx meningioma (p = 0.031), loss of the brain-tumor interface (p=0.033) and WHO grade II-III (p = 0.032).

**Conclusions:**

The resection of parasagittal and falx meningiomas in the middle third carries a high rate of postoperative morbidity and deserves perioperative planning. Alternative surgical strategies, such as preserving the brain-tumor arachnoid interface and minority residual tumors, may prevent motor decline in a subset of high-risk patients.

## Introduction

Meningiomas are the most common type of primary brain tumor derived from arachnoid cap cells. More than 90% of meningiomas are benign (WHO grade I) and grow slowly. They are mainly located on the supratentorial convexity (35%), parasagittal (20%) and falx areas (8.5%) (1, 2). Surgery is the main treatment method for meningiomas. Tumors invade the superior sagittal sinus (SSS), adhere to the cortex and are surrounded by cortical bridging veins, which increases the difficulty of surgery. When the tumor is located in the middle third of the SSS (middle third located between the coronal and lambdoid sutures) (3), it will invade the sensory-motor cortex and the SSS. The tumor is often accompanied by the middle frontal, posterior frontal, or central sulcus veins, and postoperative motor decline or even loss may occur (4). The optimal management should consider tumor-function balance, striving to achieve maximum safe resection while preserving function and improving/restoring clinical symptoms. However, several studies have indicated that the proportion of postoperative motor decline in this area is 47% for parasagittal/falx meningiomas (3), 24.7% for convexity and parasagittal meningiomas (1) and 18.6% for convexity, parasagittal and falx meningiomas (5). This seriously affects the quality of life of patients and poses a considerable challenge for surgery.

To reduce the risk of motor cortex injury, intraoperative methods such as cortical localization, electrophysiological monitoring, and neuronavigation are used to reduce the risk of cortical functional damage (6). However, studies have shown that the abovementioned techniques only have a suggestive effect during surgery when the tumor-arachnoid plane is disrupted, but at this point, cortical function has already been impaired (5). Therefore, there are uncertainties regarding the effectiveness of these methods and the surgical duration and risk of infection. It is meaningful to simply and effectively predict motor decline and understand the prognosis after motor decline.

Previous literature has highlighted the increased risk of postoperative morbidity for middle third parasagittal/falx meningioma (7, 8). However, the clinical, imaging, and pathological predictors of postoperative weakness following meningioma resection in the middle third have not been reported in large case series. In this study, we attempted to understand the rate of postoperative motor decline and identify the predictive factors for motor decline to provide guidance for subsequent treatment during the perioperative period.

## Methods

### Patient selection

**Figure 1:**
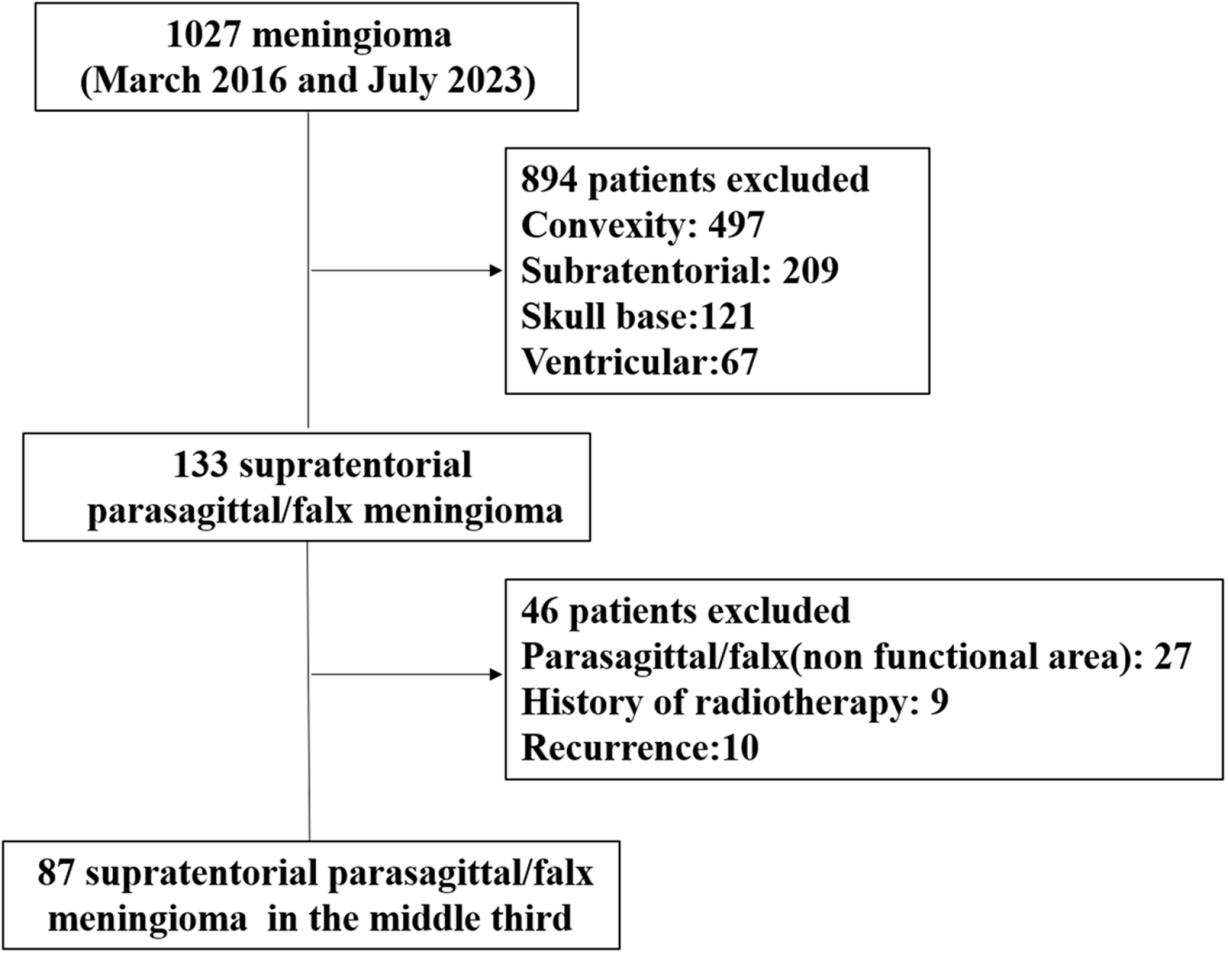
Flowchart for selection of cases in study.

A single-institution retrospective cohort study was performed on patients who underwent surgical resection at the Department of Neurosurgery, Tangdu Hospital of the Air Force Medical University (Xi’an, China), for supratentorial parasagittal/falx meningioma in the middle third SSS between March 2016 and July 2023. Patient data were collected from radiology systems and electronic medical records. The exclusion criteria for patients were as follows (**Figure 1**): (1) had a parasagittal/falx meningioma spanning the coronal or lambdoid suture, (2) had a parasagittal/falx meningioma with a history of radiotherapy, and (3) had a recurrent parasagittal/falx meningioma. All the study procedures were approved by the ethics committee of Tangdu Hospital and conducted in accordance with the guidelines of the Helsinki Declaration.

### Variables and data collection

All patient data were collected from hospital electronic medical records. Follow-up data were obtained via telephone interviews/from outpatient services. Clinical data, such as age, sex and clinical presentation (headache, dizziness, seizure, motor deficits, paresthesia or asymptomatic), were retrieved. Tumor localization was classified as parasagittal or falx. The tumor size and extent of peritumoral edema were determined based on the maximum axial diameter on gadolinium-enhanced T1-weighted and FLAIR MR images, respectively. Extent of resection: Gross total resection (GTR) was defined as Simpson grade I–III resection, and subtotal resection (STR) was defined as grade IV–V resection. The WHO grade was defined according to the 2016 WHO classification. Motor deficits evaluated through MRC (Medical Research Council) strength values. Mild motor deficits were defined as muscle strength grades 4 and 5, and severe deficits were defined as muscle strength grades 1, 2, and 3. Venous involvement (minor=cortical pial system, major =a draining vein or multiple adjacent cortical veins). The brain-tumor interface refers to the interface between the tumor and the arachnoid or pia and is defined as being preserved or lost.

### Statistical analysis

Categorical variables are presented as numbers (percentages), and differences were assessed using the chi-squared test or Fisher’s exact test, as appropriate. Cases with missing values or no follow-up information were omitted from further analysis. Univariate logistic regression analysis was used to identify independent risk factors associated with motor decline in the middle third of the SSS for parasagittal/falx meningioma resection. Risk factors with a p value less than 0.05 in the univariable regression analysis were included in the multivariable logistic regression analysis. Odds ratios (ORs) with 95% confidence intervals (CIs) were calculated. All the statistical analyses were performed using R software version 4.0 (R Core Team, R Foundation for Statistical Computing, Vienna, Austria, http://www.R-project.org/).

## Results

### Literature review

**Figure 2:**
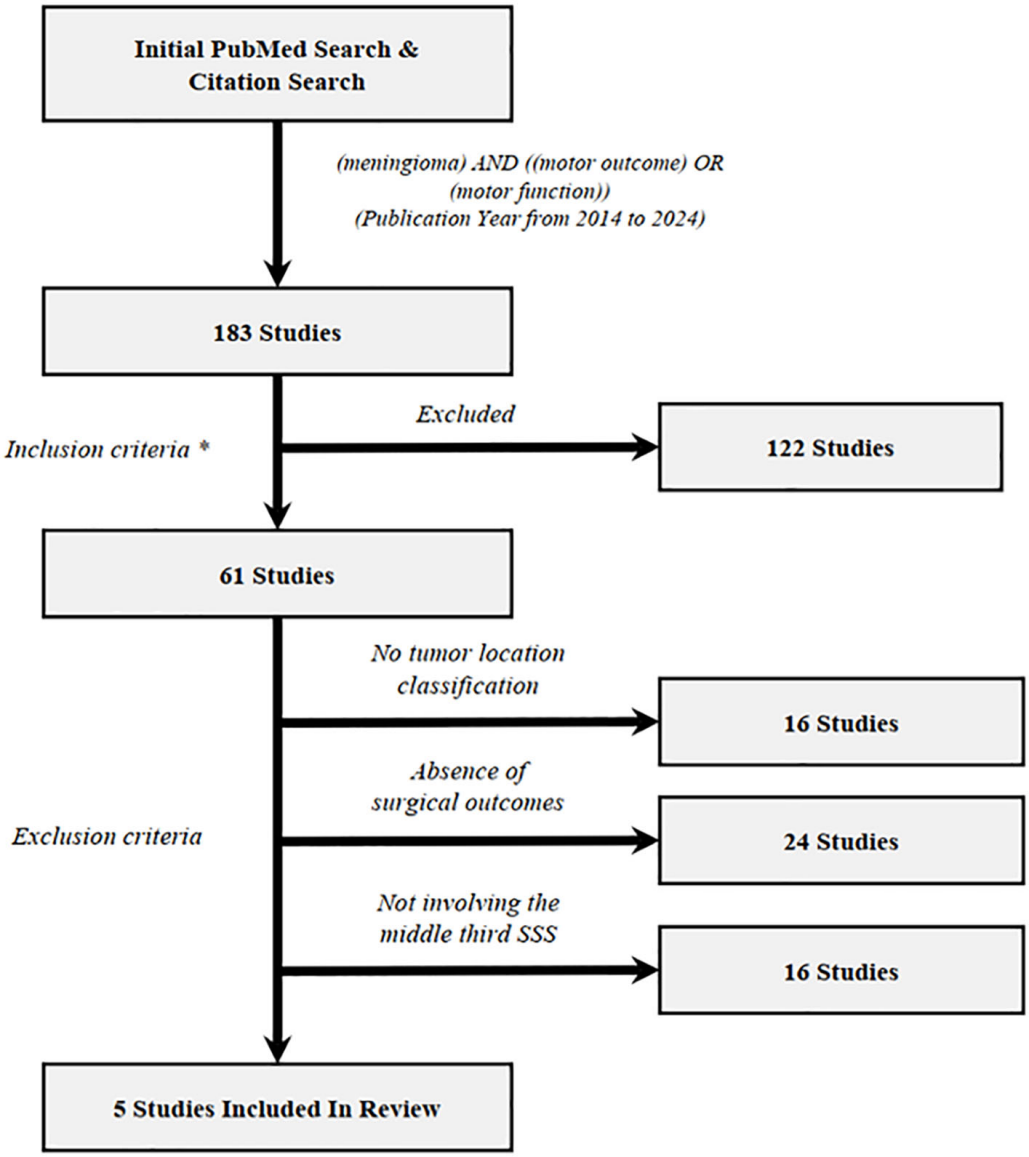
Flowchart illustrating the study selection process of the review section. Inclusion criteria (*) and exclusion criteria have been described in results section. Overall, 5 unique studies (*) meet both inclusion and exclusion criteria. (Among which 2 studies was from references in reviewed manuscripts).

181 manuscripts were identified by PubMed search and evaluated for potential inclusion. References from relevant reviews (5) were examined to identify potential manuscripts from other sources. The inclusion criterion was manuscripts with primary clinical data regarding manuscripts with primary clinical data regarding postoperative motor function in patients underwent meningioma resection. The exclusion criterion included no tumor location classification, absence of surgical outcomes and not involving the middle third SSS. To ensure data quality, only peer-reviewed, published manuscripts were included, and case reports, meeting abstracts and presentations were not enlisted. The complete process of manuscript selection, including exclusion criteria, is illustrated in a flowchart (**Figure 2**). In total, 5 manuscripts were enlisted in literature review. Among all meningioma cases, 60.8% were convexity, 21.3% were parasagittal and 17.8% were parafalcine. Preoperative and postoperative motor deficits were examined across total 202 patients underwent resection of meningioma (**Table 1**). Overall, preoperative motor deficits were observed in average 49.5% (100/202) of patients ranging between 26% to 60% across individual studies and the incidence rate of postoperative motor deterioration varied between 16% to 53% with an average of 30.2%. A total of 9, or 4.5% of patients, were identified as having permanent motor dysfunction or no recovery at the end of follow-up. Some risk factors have been described in those studies, including higher resting motor threshold (RMT) (p = 0.04) (23), lack of an intraoperative arachnoidal cleavage plane (p = 0.02) (23), preoperative motor deficit (p = 0.017) (1), minor (compared with severe) preoperative weakness (p < 0.001) (1) and preoperative embolization (p = 0.014) (1). One study suggested that parasagittal and falx meningioma involving the middle third is associated with a higher incidence of motor function deterioration either as a presenting symptom or during postoperative period, but no statistical test was provided (3).

**Table 1:** Incidence and risk factors of motor decline after meningioma resection.

Authors / Year	N	Tumor Location			Preoperative Motor Deficit	Postoperative Motor Outcome		Permanent Motor Deficit	Risk Factors of Postoperative Motor Function
		Convexity	Parasagittal	Falx		Stable	Motor Deterioration		
Tang et al. 2018 (22)	30	18 (60%)	6 (20%)	6 (20%)	14 (47%)	16 (53%)	14 (47%)	1 (3.3%) (2 yrs. follow up)	*Not specified*
Raffa et al. 2019 (23)	47	24 (51%)	16 (34%)	7 (15%)	20 (42%)	34 (72%)	13 (28%)	4 (8.5%) (3 mo. follow up)	A higher resting motor threshold (RMT) (p=0.04) Lack of an intraoperative arachnoidal cleavage plane (p=0.02)
Ottenhausen et al. 2018 (1)	89	72 (81%)	–	17 (19%)	53 (60%)	67 (73.5%)	22 (24.7%)	*Not specified*	Preoperative motor deficit (p=0.017) Minor (compared with severe) preoperative weakness (p<0.001) Preoperative embolization (p=0.014)
Elzarief et al. 2018 (3)	17	–	12 (71%)	5 (29%)	8 (47%)	8 (47%)	9 (53%)	3 (18%) (14-48 mo. follow up)	Parasagittal and falx meningioma involving the middle third? (No statistical test provided)
Coșman et al. 2020 (24)	19	9 (47.5%)	9 (47.5%)	1 (5%)	5 (26%)	16 (84%)	3 (16%)	1 (5%) (12 mo. follow up)	*Not specified*
Total	202	123 (60.8%)	43 (21.3%)	36 (17.8%)	100 (49.5%)	141 (69.8%)	61 (30.2%)	9 (4.5%)	–

### Descriptive data

In our study, most patients presented with motor deficits as their only symptom (41.4%, n = 36). A small group had only seizures (19.5%, n = 17). The remaining patients (39.1%, n = 34) were asymptomatic or had nonspecific symptoms (such as headache, dizziness, and paresthesia). Among the 36 (41.4%) patients with a preoperative motor deficit, most had mild motor weakness (72.2%, n = 26), followed by severe motor weakness (27.8%, n = 10), and the duration of deficit was chronic in 67.7% and acute in 32.3%.

Overall, the rate of postoperative motor deficits was 14.9% (13/87). Six patients experienced changes from mild motor weakness preoperatively to severe motor weakness. Seven new cases of motor weakness developed postoperatively. Among them, there were 2 patients with mild motor weakness and 5 patients with severe motor weakness. Postoperative motor decline occurred in 7 patients in the acute phase (1-3 days) and in 6 patients in the subacute phase (4-7 days). The duration of deficit was divided into short-term (10 cases, <1 months) and long-term (3 cases, ≥1months). Two patients with mild motor weakness (mild edema, MRC grade 4) had regained normal motor function after discharge. Patients with severe motor weakness (5 patients with cortical pial system injury accompanied by edema and infarction, MRC grade 3. 3 patients with collateral vein injury accompanied by edema and infarction, MRC grade 3) regained normal motor function after one month of follow-up. Among the remaining 3 patients, 1 patient suffered from a middle frontal vein injury and a secondary venous infarction. After conservative treatment, her muscle strength (MRC grade 2) returned to normal after 3 months. One patient suffered from injury to the posterior frontal vein, secondary venous bleeding (MRC grade 1), and normal muscle strength 6 months after hematoma removal. One case of central sulcus vein injury resulted in secondary venous infarction and bleeding. Muscle strength (MRC grade 2) improved slightly 1 year after hematoma removal compared to before hematoma removal (MRC grade 0).

### Postoperative risk factors related to motor decline

#### Univariate analysis

Compared with peritumoral edema <1 cm, peritumoral edema ≥1 cm was significantly more often associated with motor decline (76.9% vs. 23.1%, p = 0.034). Patients suffering from major venous involvement significantly more often exhibited postoperative motor decline than did those with nonminor venous involvement (69.2% vs. 30.8%, p =0.035). Patients suffering from falx meningioma significantly more often exhibited postoperative motor decline than did those with parasagittal meningiomas (69.2% vs. 30.8%, p = 0.013). A lost brain-tumor interface was significantly more often associated with motor decline than with a preserved brain-tumor interface (69.2% vs. 30.8%, p =0.017). Patients with WHO grades II–III significantly more often suffered from motor decline than did those with WHO grade I (69.2% vs. 30.8%, p = 0.016.) (**Tables 2**, **3**).

**Table 2:** Demographic and clinical data.

Variable	Value (%)
**No. of patients**	87
Sex	
Male	22 (25.3%)
Female	65 (74.7%)
Age	
<60	49 (56.3%)
≥60	38 (43.7%)
Histological grade	
I	79 (90.8%)
II-III	8(9.2%)
Tumor size (mm)	
<40	50 (57.5%)
≥40	37 (42.5%)
Tumor localization	
Parasagittal	55 (63.2%)
Falx	32 (36.8%)
Peritumoral edema	
<1 cm	47 (54.0%)
≥1 cm	40 (46.0%)
Sindou classification (para)	
I-III	29 (33.3%)
IV-VI	26 (29.9%)
Extent of resection	
Gross total	75 (86.2%)
Subtotal	12 (13.8%)
Venous involvement	
Minor	53 (60.9%)
Major	34 (39.1%)
Brain-tumor interface	
Preserved	54 (62.1%)
Lost	33 (37.9%)
Surgical complications	
Motor weakness	12 (14.9%)
Venous edema/infarction	8 (9.2%)
Seizure	5 (5.7%)
Paresthesia	3 (3.4%)
Hemorrhage	2 (2.1%)
Infection	2 (2.1%)

**Table 3:** Univariate analysis of motor decline in middle third SSS for parasagittal/falx meningiomas resection.

Variable	Motor decline		OR (95% CI)	*p* -Value
	(-)	(+)		
Sex				0.300
Male	17 (23.0%)	5 (38.5%)	2.09 (0.55-7.31)	
Female	57 (77.0%)	8 (61.5%)		
Age (years)				0.618
<60	43 (58.1%)	6 (46.2%)	1.60 (0.48-5.57)	
≥60	31 (41.9%)	7 (53.8%)		
Peritumoral edema				0.034
<1cm	44 (59.5%)	3 (23.1%)	4.65 (1.27-23.3)	
≥1cm	30 (40.5%)	10 (76.9%)		
Tumor size				0.986
<40mm	42 (56.8%)	8 (61.5%)	0.83 (0.23-2.79)	
≥40mm	32 (43.2%)	5 (38.5%)		
Venous involvement				0.035
Minor	49 (66.2%)	4 (30.8%)	4.25 (1.23-17.6)	
Major	25 (33.8%)	9 (69.2%)		
Origin				0.013
Parasagittal	51 (68.9%)	4 (30.8%)	4.80 (1.38-20.0)	
Falx	23 (31.1%)	9 (69.2%)		
Extent of resection				0.378
Gross total	65 (87.8%)	10 (76.9%)	2.19 (0.41-9.18)	
Subtotal	9 (12.2%)	3 (23.1%)		
Brain-tumor interface				0.017
Preserved	50 (67.6%)	4 (30.8%)	4.51 (1.30-18.7)	
Lost	24 (32.4%)	9 (69.2%)		
Histological grade				0.016
WHO I	70 (94.6%)	9 (69.2%)	7.46 (1.46-38.9)	
WHO II-III	4 (5.41%)	4 (30.8%)		

#### Multivariate analysis

**Figure 3:**
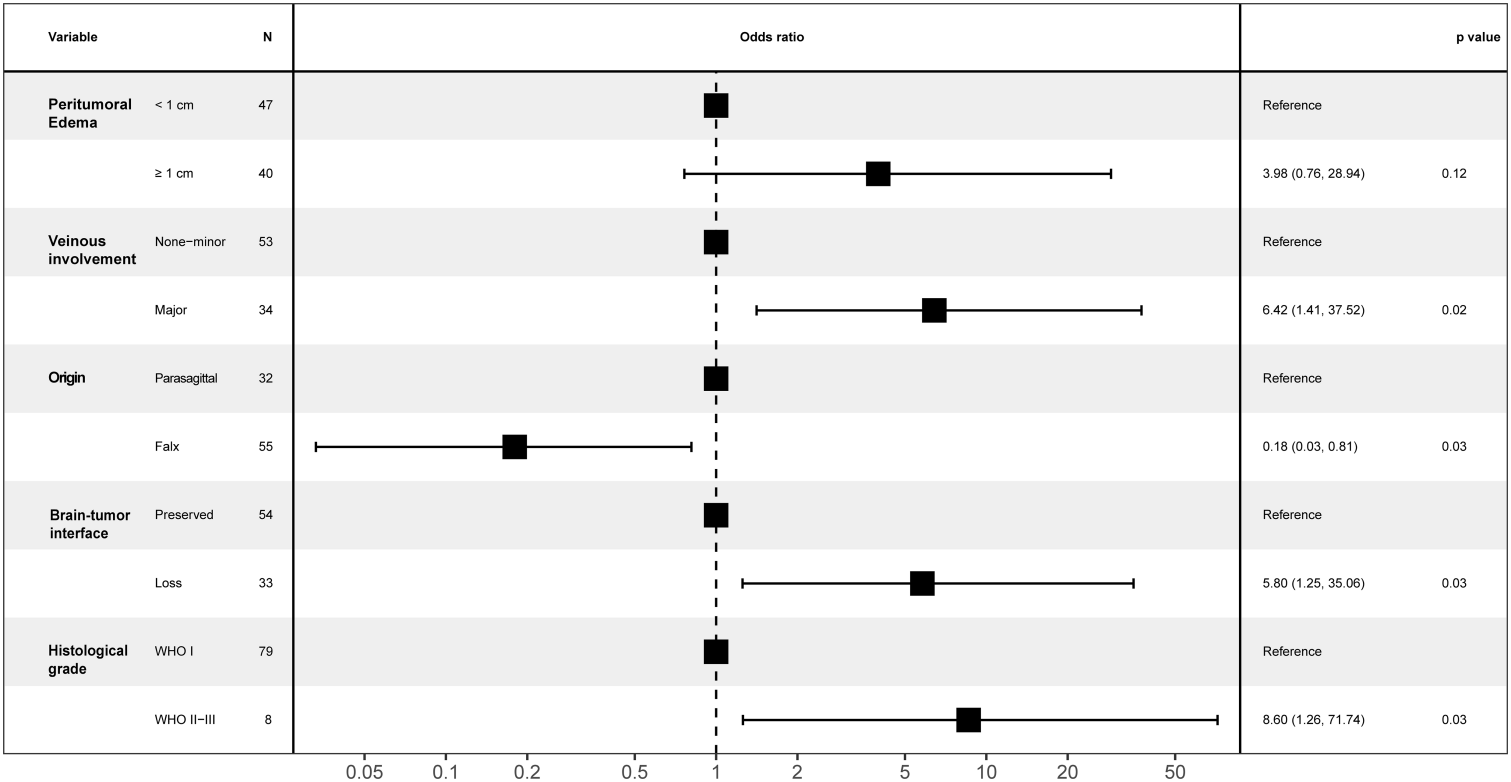
Forest plot for motor decline following parasagittal/falx meningioma resection in the middle third SSS.

**Figure 4:**
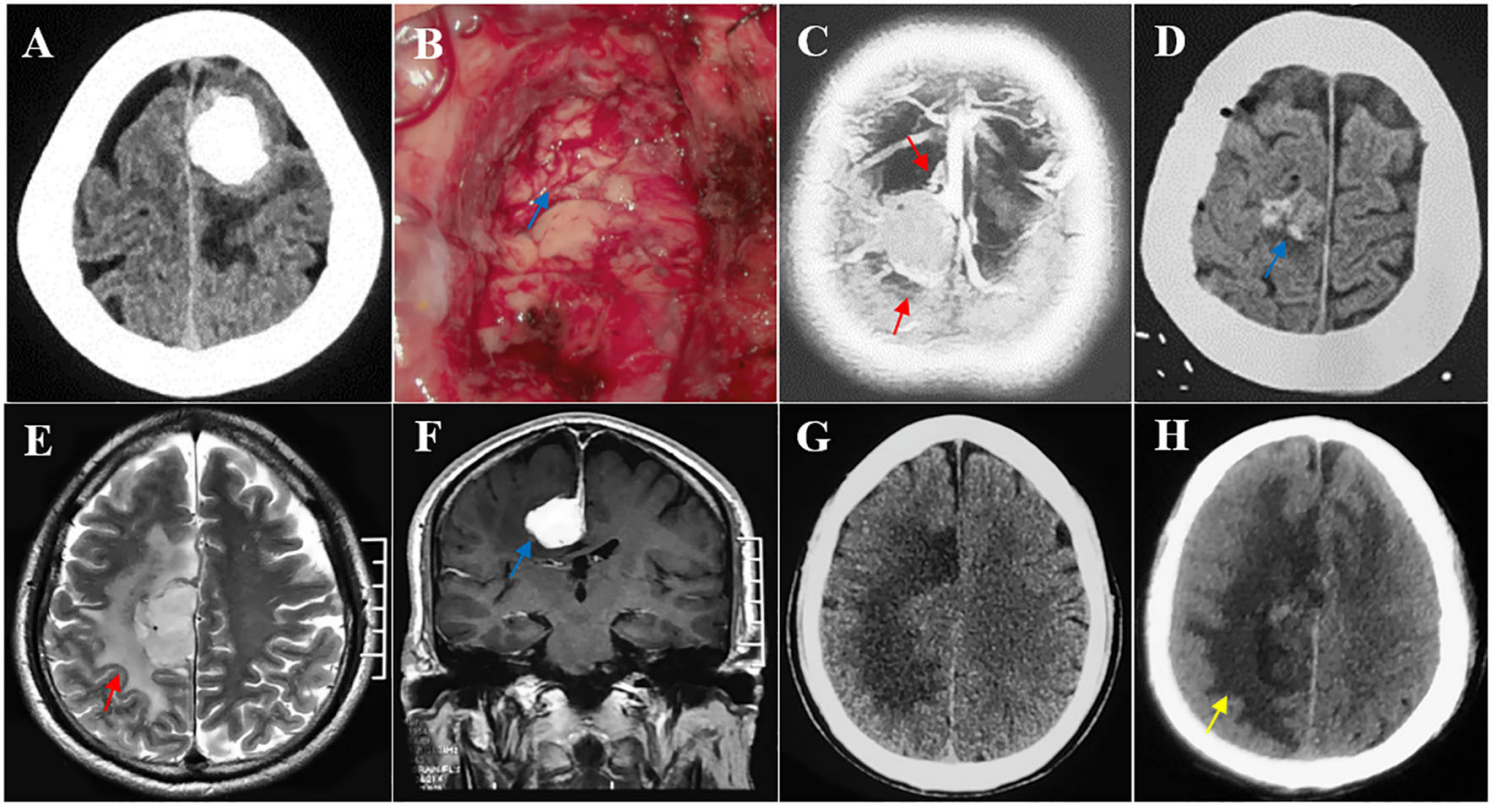
Multiple risk factors leading to postoperative motor decline after parasagittal/falx meningioma resection in the middle third SSS. **(A, B)** The tumor invading the pia mater was completely removed, and the brain-tumor interface was lost intraoperatively (blue arrow). Pathological testing revealed an atypical meningioma. **(C, D)** Multiple peritumoral veins (red arrow) and postoperative minor hemorrhage (blue arrow). **(E–H)** A deep falx meningioma (blue arrow) with obvious peritumoral edema (red arrow) and postoperative edema expanded (yellow arrow) compared with its preoperative condition.

We performed a multivariate logistic regression analysis to identify the potential predictors of postoperative motor decline in the middle third of the SSS for meningioma resection. Major venous involvement (p = 0.022, OR 6.42, 95% CI 1.29-31.7), falx meningioma (p = 0.031, OR 0.18, 95% CI 0.04-0.84), a lost brain-tumor interface (p=0.033, OR 5.79, 95% CI 1.15-29.3) and WHO grade II-III (p = 0.032, OR 8.59, 95% CI 1.21-61.2) were identified as the only independent and significant predictors of postoperative motor decline (**Figures 3**, **4**).

## Discussion

Surgical treatment of tumors near the eloquent area of the brain remains a challenge and is associated with a high incidence of postoperative neurological deficits, resulting in a negative impact on quality of life. Although brain tumors, especially gliomas, are the subject of many studies in the literature (9, 10), there is less debate on the issue of middle third parasagittal/falx meningiomas. Most studies (11–13) include all meningiomas of the brain convexity, parasagittal and falx regions without focusing on the middle third SSS location and providing separate data on parasagittal/falx meningiomas.

To our knowledge, this is the largest study to reveal the neurological outcomes of middle third parasagittal/falx meningioma resection and the risk factors for postoperative motor weakness. Our univariate data analysis revealed risk factors, including a lost brain-tumor interface, major venous involvement, peritumoral edema, falx location, and malignant meningioma. Further multivariate analysis revealed that peritumoral edema is not a risk factor for postoperative motor decline. Although there have been reports that peritumoral edema is a risk factor for postoperative decline in the motor area, the data include convexity or recurrent meningiomas (1, 5). Further large-scale validation is still needed in the future.

Postoperative motor decline is characterized by transient edema, ischemic or hemorrhagic infarction, or permanent necrosis of the motor cortex. As an extracerebral tumor, a meningioma has a typical brain-tumor interface due to its biological characteristics. Preoperative motor decline is mainly caused by the mass effect of the meningioma, which leads to cortical edema, causing transient functional impairment. In theory, if the tumor-brain interface is not disrupted during surgery, the cerebral cortex will not be damaged, and motor function will not be affected. Patients with loss of the arachnoid cleavage plane exhibit tumor-pial adhesion and cortical invasion (5). According to several anatomical studies, the initial segment of pyramidal cells is located approximately 1.3 millimeters below the cortical surface (6). Therefore, pia mater adhesion does not damage motor cells. This is very important for microsurgical operations at the brain-tumor interface. While maximizing tumor resection, a very thin tumor layer remains, and the brain-tumor interface at every point on the surface of the tumor must be carefully studied. If it is easy to perform a gentle dissection, the excision can be completed. If the adhesion is hard, placing residual 1-2 mm tumors on the cerebral cortex surface is suggested. Increasing evidence suggests that brain tissue invasion and subtotal resection do not increase the risk of meningioma (WHO grade I) recurrence; only high-grade meningiomas (WHO grade II-III) are significantly associated with an increased recurrence risk (14, 15), and the possibility of recurrence can be reduced through radiotherapy (16). Therefore, the integrity of the tumor-brain interface is highly important. Bipolar coagulation at the brain-tumor interface should be avoided intraoperatively because it can cause vascular damage, leading to cortical edema and infarction.

The peritumoral veins in the middle third of the SSS are mainly the middle frontal, posterior frontal, and central sulcus veins. Once venous injury occurs during surgery, it leads to venous reflux obstruction, secondary venous hypertension, venous edema/infarction and cortical dysfunction, which manifests as motor decline, seizures, and hemorrhagic infarction (12, 13, 17, 18). Surgical morbidity and mortality in patients with parasagittal and falx meningiomas are mainly caused by venous damage resulting from drainage impairment, and a venous infarction or hemorrhage is the fundamental cause of other postoperative complications. Venous infarction was most commonly associated with a falx meningioma, followed by a parasagittal infarction. Multiple studies have shown that the average incidence of venous infarction after parasagittal and falx meningioma resection is 7.2% and 9.4%, respectively (12, 13, 19–21). A high grade meningioma, due to its biological characteristics, is invasive, damages the blood−brain barrier and the pia mater, and has obvious adhesion to brain tissue. Intraoperative pial-arteriovenous system injury leads to cortical edema and ischemia, as well as motor weakness, which is also caused by venous infarction (21).

Postoperative motor decline can be divided into the acute phase (1-3 days), subacute phase (3-14 days), and late phase (>14 days). The acute phase is mainly caused by a venous infarction, which leads to acute cortical dysfunction and severe motor decline immediately after surgery. During the subacute phase, motor decline is caused by intraoperative cortical traction to increase brain edema and ischemia, resulting in progressive deterioration of motor function, which gradually improves within one month of conservative treatment. If there are no external factors (such as seizures) in the later stage, the degree of motor decline is generally not aggravated, and the patient is in a stable recovery period. According to our one-year follow-up of case data, the degree of motor impairment in patients improved to varying degrees, with some improvements being significant. However, intraoperative damage to major veins (posterior frontal and central sulcus veins) can lead to a postoperative venous infarction or hemorrhage, resulting in severe motor decline, limited recovery and even the inability to recover in the later stage (18). Therefore, we believe that the ability to recover motor function mainly depends on the degree of intraoperative damage to the veins in the motor cortex region.

### Study limitations

There are several limitations that should be noted in our study. 1. The sample size is small, and there may be bias in the statistical results. 2. Short follow-up time leads to inaccurate prognosis results. 3. Due to limitations in hospital conditions and issues with the integrity of tumor samples, pathological examinations of tumors cannot be updated with the latest WHO pathological classification in a timely manner, which may result in some results being biased.

## Conclusions

Parasagittal and falx meningiomas in the middle third, with a lost brain–tumor interface, major venous involvement, and a falx location, as well as high-grade meningiomas are associated with a higher risk of postoperative worsening of motor function. These findings are extremely valuable for patient counseling and surgical decision-making. Therefore, a comprehensive evaluation was conducted based on patient symptoms and imaging data before surgery, and intraoperative microsurgical operations were performed to preserve anatomical integrity and reduce cortical damage.

## Data Availability

The data analyzed in this study is subject to the following licenses/restrictions: The raw/processed data required to reproduce these findings cannot be shared at this time as the data also forms part of an ongoing study. Requests to access these datasets should be directed to sxcaiqing@163.com.
